# Deciphering Structural Intermediates and Genotoxic Fibrillar Aggregates of Albumins: A Molecular Mechanism Underlying for Degenerative Diseases

**DOI:** 10.1371/journal.pone.0054061

**Published:** 2013-01-14

**Authors:** Aabgeena Naeem, Samreen Amani

**Affiliations:** Department of Biochemistry, Faculty of Life Science, Aligarh Muslim University, Aligarh, Uttar Pradesh, India; Russian Academy of Sciences, Institute for Biological Instrumentation, Russian Federation

## Abstract

The misfolding and aggregation of proteins is involved in some of the most prevalent neurodegenerative disorders. The importance of human serum albumin (HSA) stems from the fact that it is involved in bio-regulatory and transport phenomena. Here the effect of acetonitrile (ACN) on the conformational stability of HSA and by comparison, ovalbumin (OVA) has been evaluated in the presence and absence of NaCl. The results show the presence of significant amount of secondary structure in HSA at 70% ACN and in OVA at 50% ACN, as evident from far-UV Circular Dichroism (CD) and Attenuated Total Reflection Fourier transformed infra red spectroscopy (ATR-FTIR). Tryptophan and 8-Anilino-1-Naphthalene-Sulphonic acid (ANS) fluorescence indicate altered tryptophan environment and high ANS binding suggesting a compact “molten globule”-like conformation with enhanced exposure of hydrophobic surface area. However, in presence of NaCl no intermediate state was observed. Detection of aggregates in HSA and OVA was possible at 90% ACN. Aggregates possess extensive β-sheet structure as revealed by far-UV CD and ATR-FTIR. These aggregates exhibit increase Thioflavin T (Th T) fluorescence with a red shift of Congo red (CR) absorption spectrum. X-ray diffraction (XRD) and Scanning Electron Microscopy (SEM) analysis confirmed the presence of fibrillar aggregates. Single cell gel electrophoresis (SCGE) assay of these fibrillar aggregates showed the DNA damage resulting in cell necrosis confirming their genotoxic nature. Some proteins not related to any human disease form fibrils in vitro. In the present study ACN gives access to a model system to study the process of aggregation.

## Introduction

The process by which a linear sequence of amino acids folds into a discrete and functional three-dimensional protein is the most fundamental and universal example of biological self assembly. Although the code that governs folding remains a mystery, the primary sequence is subject to evolutionary pressure for adjusting the folding rate and product stability according to physiological needs. The failure of a protein to fold correctly leads to a functional deficit. The concept that amino acids sequences determine protein conformation is now fully accepted [1]–[2]. The ability of the side chains to form the tight and specific interactions like that of a native protein is an important final step in the protein folding pathway [3]. Nonspecific interactions among the side chains or inappropriately exposed hydrophobic surfaces of incompletely folded polypeptides result in coagulation, most widely viewed as protein aggregation. Thus, production of misfolded or denatured proteins is potentially deleterious to cells because of their ability to co-aggregate with and thereby trap unrelated cellular proteins that may transiently display complementary surfaces [4]–[5]. Abnormal interactions has been proposed to underlie the toxicity associated with protein aggregates in many neurodegenerative disorders like Alzheimers, Parkinsons, Creutzfeldt-Jakob Disease, etc [6].

The aggregation process occurs in competition with normal folding process [7]. A range of proteins not related to any human disease have been found to form fibrils in vitro under mildly denaturing conditions which are similar in characteristic to those proteins that are disease-associated [8]. Small globular proteins that act in this manner serve as a model system for checking the general characteristics of amyloid and for understanding the overall significance of aggregation in cell biology [9]–[10]. This observation has led to the suggestion that the ability to form amyloid fibrils is a common phenomenon and a generic property of polypeptide chains. A considerable number of proteins, including several that adopt α-helical structures under native conditions such as myoglobin and cytochrome c-_552_, have been shown to form amyloid fibrils in vitro, provided appropriate conditions are selected [11]–[12]. The ability of rational design of conditions promoting aggregation has implications for understanding the origin of amyloid formation in vivo from a wide range of proteins. This gives access to a large number of model systems with which to study the process of fibril formation in more detail. This study capitalizes on Ovalbumin (OVA) and human serum albumin (HSA) as models for protein aggregation as evaluated by a number of complementary techniques. OVA is a globular protein composed of 385 amino acids and is a member of the non-inhibitory serpin superfamily [13]. It is a useful model system to study helix/sheet transitions, because it represents a protein that has almost equal proportions of α-helix and β-sheet (30.6 and 31.4%, respectively). An erroneous transition from α-helix to β-sheet structures has fatal consequences in prion and other amylogenic diseases [14]. HSA, on the other hand, is a primarily α-helical protein whose 585 amino acid structure is divided into multiple domains [15]. In view of its independently folding domains, involvement of inter-domain interactions in the folding mechanism, and propensity to aggregate in vitro, HSA is recognized as a good model for folding as well as aggregation studies. HSA participates in the control of osmotic pressure in blood. It binds various metal ions and takes part in transport and storage of different fatty acids [16]. It also binds bilirubin, steroids, and amino acids. This unique property enables HSA to fulfill a fundamental role as a universal biological carrier and reservoir throughout the human body. Also, deficiency of serum albumins is known to cause a number of diseases [17] including Wiskott-Aldrich, Nephrotic syndrome etc.

In the present study, Acetonitrile (ACN) is used as model solvent for evaluating its effect on HSA and OVA as model proteins. ACN is mainly used in pharmaceutical industries for the assessment of potency as well as impurity levels of drugs and to extract fatty acids from animal and vegetable oils. It is also found in drinking water as chlorinated and brominated halo-acetonitriles contaminants which are formed during chlorine disinfection [18]. The general population is exposed to ACN by inhalation resulting in development of disorders like chronic obstructive pulmonary disease, impaired blood clotting, abnormal kidney and liver function [19]. However, in this present study, we report that appropriately designed controlled solvent conditions can promote the formation of intermediates and fibrils aggregates providing the opportunity to investigate the molecular basis of aggregation. Here we aimed at developing an experimental, in vitro model for aggregation studies (using ACN as an organic solvent). The solvent ACN, at high concentration, destabilized the native globular fold of HSA as well as OVA resulting in aggregation of proteins, in which non-covalent interactions still remain favorable. ACN is observed to induce amyloid-like features in HSA which resembles proteins involved in conformational diseases.

## Materials and Methods

HSA (A9511, ≥ 97% pure) and OVA (A5503, ≥ 98% pure) were purchased from Sigma (US). Purity of HSA and OVA was checked by sodium dodecyl sulphate–polyacrylamide gel electrophoresis. ACN was purchased from SRL (Mumbai, India) without further purification; 8-Anilino-1-Naphthalene-Sulphonic acid (ANS), Thioflavin T (Th T) and Congo red (CR) were bought from Sigma (St. Louis, MO, USA).

The stock solution of protein (5 mg/ml) was prepared in 20 mM sodium phosphate buffer of pH 7.2 and it was then dialyzed in the same buffer. The concentration of native protein in 20 mM sodium phosphate buffer, pH 7.2, was determined from extinction co-efficient of 6.99 and 5.30 A/1%/1 cm, for OVA and HSA respectively by UV absorption at 280 nm on a Shimadzu UV-1700 spectrometer.

### Effect of ACN on HSA and OVA

Samples of HSA and OVA were prepared separately with varying concentration of ACN i.e. 0% to 90% at pH 7.2 and then these samples were incubated for 4 hours before performing spectroscopic measurements. All the measurements were carried out at room temperature. Three replicates for each set were analyzed for the results.

### Intrinsic Fluorescence Measurements

The fluorescence spectra were recorded on a Shimadzu RF-5301 spectrofluorophotometer (Tokyo, Japan) in a 10 mm path length quartz cell. The excitation wavelength was 295 nm and the emission was recorded in the range of 300–400 nm [20]. The final concentration of protein in OVA samples was 4.44 µM and 3.03 µM in HSA samples.

### Acrylamide Quenching Studies

In the acrylamide-quenching experiments, aliquots of 5 M acrylamide stock solution were added to a protein stock solution (15 µM) to achieve the desired acrylamide concentration. Excitation was set at 295 nm in order to excite only tryptophan fluorescence, and emission was recorded in the range of 300–400 nm. The decrease in fluorescence intensity at λ_max_ was analyzed according to the Stern–Volmer equation [21]:
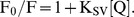
where F_0_ and F are the fluorescence intensities at an appropriate wavelength in the absence and presence of acrylamide, respectively, K_SV_ is the Stern–Volmer constant for the collisional quenching process, and [Q] is the concentration of the quencher.

### ANS Fluorescence Measurements

ANS binding was measured by fluorescence emission spectra with excitation at 380 nm and emission was recorded from 400 to 600 nm [22]. Typically, ANS concentration was 100 molar excess of protein concentration [23]–[25] and protein concentration was in the vicinity of 4.44 µM for OVA and 3.03 µM for HSA.

### Attenuated Total Reflection Fourier Transformed Infra Red Spectroscopy (ATR-FTIR)

ATR-FTIR spectra were recorded with an Interspec 2020 FTIR spectrometer in deuterated water in the amide I region in the range of 1720 to 1580 cm^−1^. Protein concentration was 44.4 for OVA and 30.3 µM for HSA. The scanning wave number was from 1000–4000 cm^−1^ [9].

### Circular Dichroism (CD) Measurements

CD was measured with a JASCO J-810 spectropolarimeter calibrated with ammonium D-10-camphorsulfonate. Cell of path lengths 0.1 was used for scanning between 250-200 nm. For signal to noise ratio, each spectrum was the average of 4 scans. Base lining and analysis were done using Jasco J-720 software. Protein concentration for the scans was 4.44 µM for OVA and 3.03 µM for HSA.

### Rayleigh Scattering Measurements

Rayleigh scattering measurement was performed on Shimadzu RF-5301 spectrofluorophotometer (Tokyo, Japan) in a 1 cm path length quartz cell. The excitation wavelength was set at 350 nm and emission range was 300–400 nm. Both excitation and emission slit width are fixed at 5 nm. Fluorescence intensities at 350 nm were plotted. The final concentration of OVA and HSA were 4.44 and 3.03 µM.

### Thioflavin T Assay

Fluorescence spectra were recorded with a Shimadzu RF-5301 spectrofluorophotometer in a 10 mm path length quartz cell. The excitation wavelength was 440 nm and the emission was recorded from 450 to 600 nm. For aggregation studies Th T was added to the samples incubated for different time period i.e. 4, 6, 8, 12, 24 and 48 hours and then spectra were recorded. Final concentration of protein was 4.44 µM and 3.03 µM for OVA and HSA respectively while the concentration of Th T was 20 µM. Th T was prepared in 20 mM sodium phosphate buffer, pH 7.2 [26].

### Congo Red Assay

Absorption spectra were recorded in the range between 400–700 nm on Shimadzu UV-1700 Spectrophotometer by using cuvette having path length 1 cm. Samples were prepared in presence of ACN with protein concentration of 0.4 mg/ml and incubated for 4 hours. 60 µl of each sample is added to 440 µl of a solution containing 20 µM CR in 20 mM phosphate buffer. After 2–3 min of equilibration, absorbance was recorded [27].

### Dynamic Light Scattering (DLS) Measurements

DLS studies were carried out on DynaPro–TC–04 dynamic light scattering equipment (Protein Solutions, Wyatt Technology, Santa Barbara, CA) equipped with temperature-controlled micro-sampler. OVA and HSA were taken in a concentration of 44.4 and 30.3 µM for the analysis. All the solutions were spun at 10,000 rpm for 15 min, prior to scanning, and first filtered through microfilter (Millipore Millex-HV hydrophilic PVDF) having a pore size of 0.45 µm followed by filteration using 0.22 µm pore sized filter. Measured size was presented as the average value of 50 runs. Dynamics 6.10.0.10 software at optimized resolution was used for data analysis. The mean hydrodynamic radius (R_h_) and Polydispersity (P*d*) were estimated on the basis of an autocorrelation analysis of scattered light intensity based on translational diffusion coefficient, by Stokes–Einstein equation:
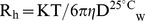
where R_h_ is the hydrodynamic radius, k is the Boltzman’s constant, T is the absolute temperature, η is the viscosity of water and D^25°C^_w_ is the translational diffusion coefficient.

### X-ray Diffraction (XRD) Studies

Aggregated HSA was prepared by taking 10 mg/ml of HSA in 90% ACN and incubating it for 4 hrs. After locking the conformation, the ACN treated HSA was air dried to remove ACN. The studies were carried out using a Rigaku X-ray- powder diffractometer with Cu anode (Cu- Kα radiation λ = 1.54186 Å) in the range of 20°≤2θ≤80° at 30 kV. The peak positions, intensities, widths and shapes all provide important information about the structure of the material.

### Single Cell Gel Electrophoresis (SCGE) of the Aggregated HSA

Isolated lymphocytes were exposed to 50 µgm of HSA aggregates (after removing the ACN by air drying) in a total reaction volume of 1.0 ml of 20 mM phosphate buffer pH 7.2. Incubation was performed at 37°C for 1 h. After incubation, the reaction mixture was centrifuged at 716.8 g, the supernatant was discarded and pelleted lymphocytes were resuspended in 100 µL of PBS and processed further for SCGE assay. SCGE assay of protein aggregates was performed under alkaline conditions by the procedure of Khan et al. [28].

### Scanning Electron Microscopy (SEM) Analysis

Aggregates were prepared by taking 10 mg/ml of HSA in 90% ACN and incubating it for 4 hours. SEM analysis of the surface and cross-section of air dried samples of HSA aggregate was performed with JSM-6510 LV scanning electron microscope (JEOL, Japan). The samples were mounted on a carbon tape coated stainless steel grids operating on an accelerating voltage of 10 and 12 kV and in low vacuum condition.

## Results and Discussion

### Intrinsic Fluorescence

**Figure 1:**
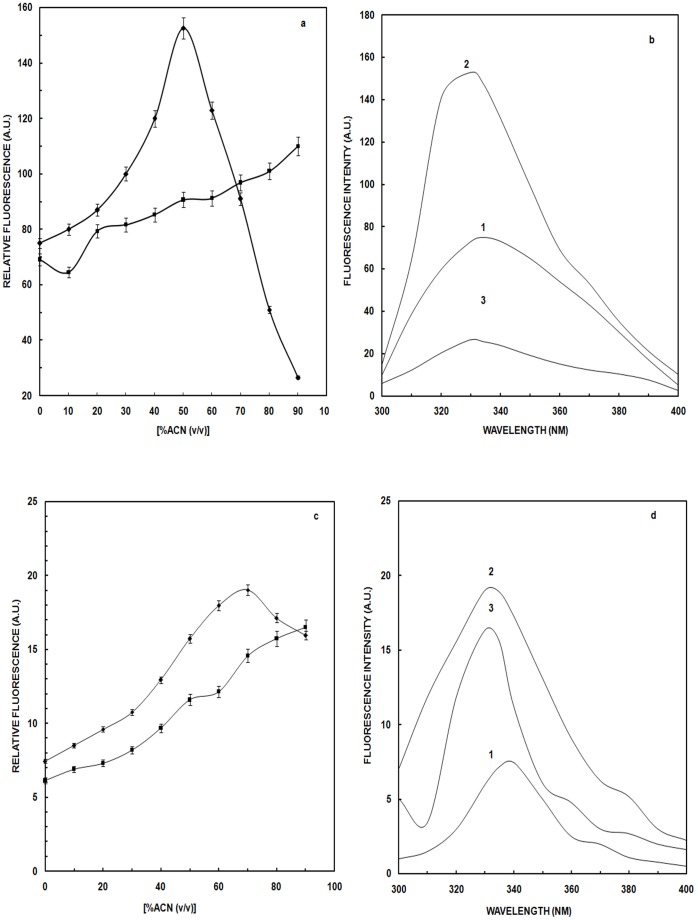
Intrinsic Fluorescence studies. (a) Relative intrinsic fluorescence intensity of OVA in absence (▪) and presence (♦) of NaCl as a function of increasing concentration of ACN, (b) Tryptophan fluorescence emission spectra of native OVA in 20 mM sodium phosphate buffer, pH 7.2 (curve 1); curves 2 and 3 represent OVA at 50% and 90% ACN respectively. OVA concentration was 4.44 µM and the path length was 1 cm. (c) Relative intrinsic fluorescence intensity of HSA in absence (▪) and presence (♦) of NaCl as a function of increasing concentration of ACN, (d) Tryptophan fluorescence emission spectra of native HSA in 20 mM sodium phosphate buffer, pH 7.2 (curve 1); curves 2 and 3 represent HSA at 70% and 90% ACN. HSA concentration was 3.03 µM and the path length was 1 cm. The fluorescence intensity measurement was carried out at an excitation wavelength of 280 nm.

Intrinsic fluorescence of a protein (primarily due to the aggregate behavior of its tryptophan and tyrosine residues) is a sensitive reporter of protein conformational changes. OVA have three tryptophan residues (148, 184, and 267) and eight tyrosine residues [29]. We evaluated the relative fluorescence of OVA as a function of ACN concentration in the presence or absence of 0.9% NaCl (Figure 1A). In the absence of NaCl, a bipartite response was observed with fluorescence intensity approaching a maximum in the presence of 50% v/v ACN *(Unless otherwise indicated all ACN concentrations are present in percent v/v)* and decreasing thereafter by a factor of eight at 90%. The data were indicative of an intermediate appearing during the ACN-induced structural transition of OVA. A drop beyond 50% is possibly due to internalization of all three tryptophan residues owing to intermolecular protein-protein interaction probably due to the formation of aggregates. Conversely, in presence of NaCl, there was a continuous albeit modest increase in fluorescence intensity, indicating that the ions substantially limit the ability of ACN to induce structural changes in OVA. Salts often impart greater stability to proteins and may explain this delay in OVA unfolding. In the absence of NaCl, the transition is a two-step, three-state process [30]. Acetonitrile alone at various concentrations (10–90%) is also monitored and here we have reported the subtracted spectra. Figure 1B depicts the emission fluorescence spectra of OVA in presence of ACN. Addition of 50% ACN (curve 2) results in ∼50% increase in fluorescence intensity with a blue shift of 4 nm (from 334 to 330 nm) compared to native (curve 1) indicating internalization of surface exposed Trp residues in non-polar environment. On further addition of ACN, up to 90% (curve 3), a progressive reduction in tryptophan fluorescence intensity relative to the intermediate state at 50% [∼34% fluorescence intensity of native (curve 1)] was observed. This further decrease in fluorescence intensity was a result of cross-linking of individual OVA molecules due to aggregation.

HSA has a single tryptophanyl residue, Trp 214, located at the interface between domains II and III. Since the position of wavelength maximum of emission spectrum depends on the properties of the environment of this one tryptophanyl residue, small changes in the conformation of HSA are anticipated to produce marked changes in fluorescence properties. Figure 1C shows the relative fluorescence intensity for HSA incubated with different concentration of ACN in the absence and presence of 0.9% NaCl. Initially there is increase in tryptophan intensity on addition of ACN up to 70% in absence of NaCl, followed by a decline in fluorescence up to 90% ACN. We suggest that the observed drop in fluorescence intensity after 70% ACN addition, could be due to either (i) exposure of tryptophan to the solvent and/or (ii) quenching of tryptophan intensity due to an increase in the number of phenylalanine, histidine, and disulfide residues in the proximity of tryptophan that quenches the emission upon aggregation mediated by both electrostatic and hydrophobic interactions. In presence of NaCl, a continuous increase in fluorescence intensity was observed up to 90% ACN, indicating no intermediate was present. On increasing ACN concentration up to 70%, we observed a blue shift (340-330 nm) and enhancement in fluorescence intensity of the HSA (Figure 1D) [31]. On increasing the ACN concentration to 90% (curve 3), a red shift of 6 nm and decrease in fluorescence intensity was observed relative to that obtained in 70% ACN. However, as compared to native HSA (curve 1), 90% ACN (curve 3) induces a blue shift of 4 nm (i.e. λ_max_ at 336 nm) along with an increase in the tryptophan fluorescence of the protein. Juarez et al. have reported a similar blue shift in HSA upon aggregation [13].

### Acrlyamide Quenching

**Figure 2:**
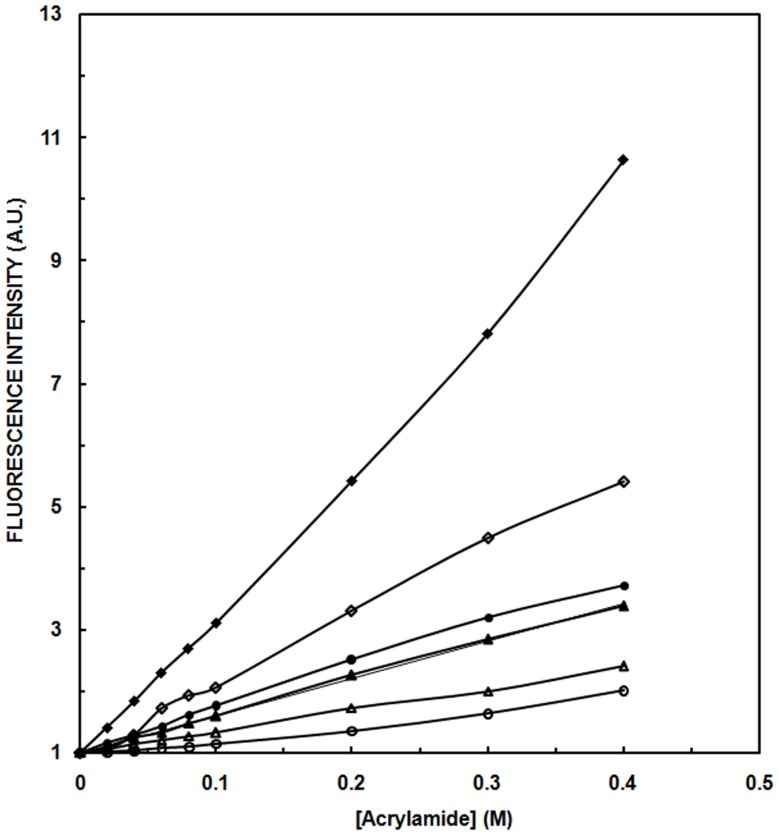
Stern-Volmer plots for acrylamide quenching of tryptophan fluorescence of albumins. Native OVA (•); HSA (▴) and NATA alone (♦) at pH 7 in presence of varying concentration of quencher. 90% ACN incubated OVA (○); HSA (**▵**) and NATA (**◊**). Values shown are the ratios of fluorescence in the absence of acrylamide (F_0_) to the fluorescence at the given concentration of quencher (F). Protein concentration for OVA was 4.44 and for HSA was 3.03 µM. Path length for the study was 1 cm and the excitation wavelength was 295 nm.

To eliminate the likelihood of fluorescence quenching owing to tryptophan residues, albumins were subjected to quenching by non-ionic molecules of acrylamide [32] with varying concentration of ACN (0–90%). Stern-Volmer plots of free and unbound model compound NATA and tryptophan residues of OVA and HSA in the presence of ACN (0–90%) has been shown in Figure 2. Acrylamide quenching of Tryptophan analogue NATA alone shows maximum Stern-Volmer constant (K_sv_) value indicating maximum quenching (Table 1). Less quenching of OVA and HSA by acrylamide in the presence of 90% ACN results in the blue shift of λ_max_ from 334 to 328 nm and from 340 to 325 nm respectively. This suggests the possibility of different availability of tryptophan residue to the quencher probably due to aggregation of albumins at 90% ACN. Acrylamide, being a hydrophilic dye, does not penetrate to the hydrophobic core of aggregates and shows less quenching [33]. Acrylamide quenching results a decrease in K_sv_ with aggregation of protein. This decrease may be due to decreased accessibility of tryptophan to solvent owing to aggregation. OVA has a larger K_sv_ value relative to HSA implicating higher propensity of later to form aggregates.

**Table 1:** Acrylamide-quenching parameters of HSA in different states.

S.No.	Subjects	K_sv_(M^−1^)	Wavelength (nm)
**1**	Native HAS	6.089	340
**2**	Native OVA	6.975	334
**3**	HSA+90% CAN	2.503	325
**4**	OVA+90% CAN	3.505	328
**5**	NATA	23.8	348
**6**	NATA+90%ACN	11.43	362

The excitation wavelength was 295 nm.

### Extrinsic Fluorescence

**Figure 3:**
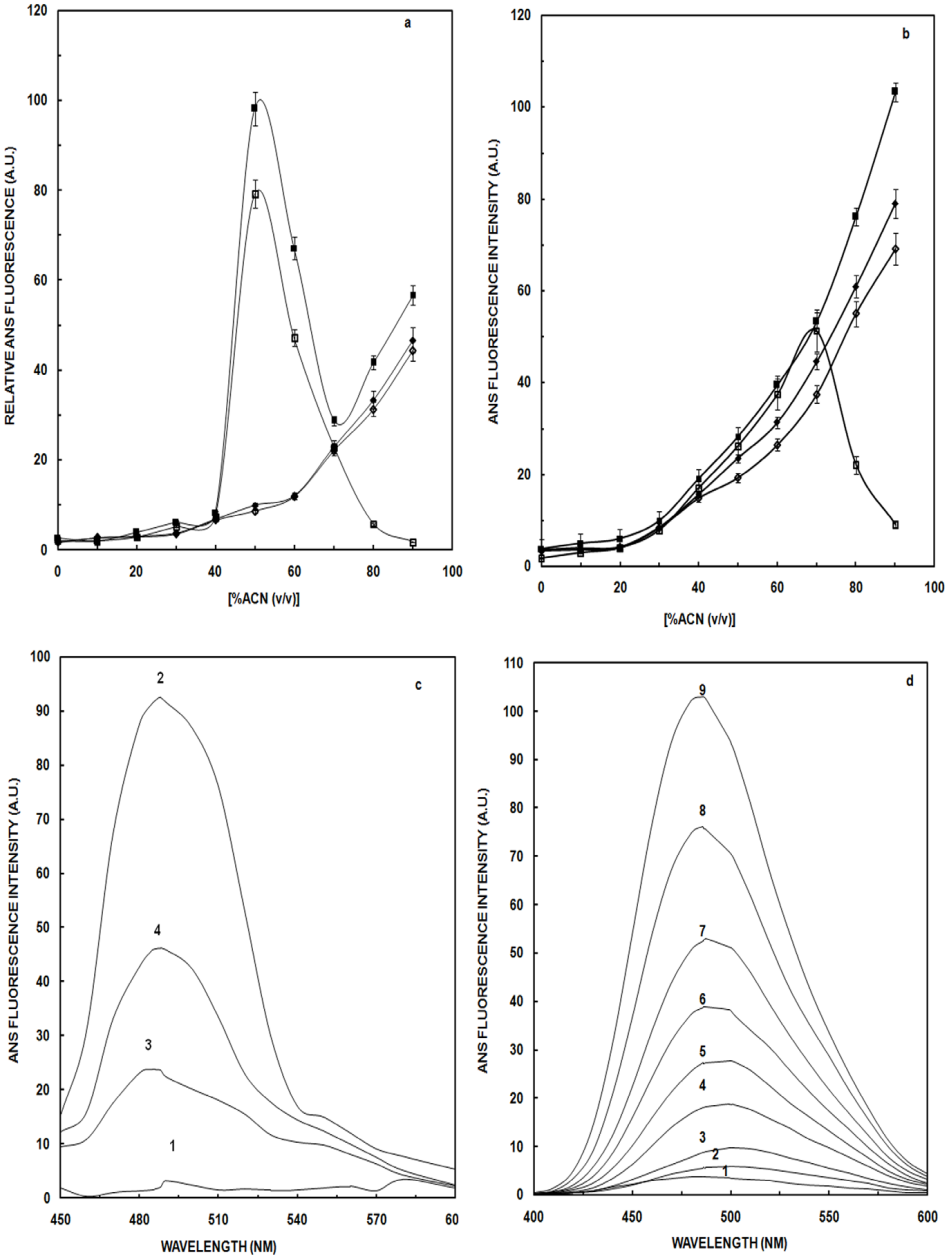
ANS Fluorescence studies. (a) Relative ANS fluorescence of OVA in absence (▪) and presence (♦) of NaCl before centrifugation and in absence (□) and presence of NaCl (⋄) after centrifugation as a function of increasing concentration of ACN; (b) Relative ANS fluorescence of HSA in absence (▪) and presence (♦) of NaCl before centrifugation and in absence (□) and presence of NaCl (⋄) after centrifugation as a function of increasing concentration of ACN. (c) ANS fluorescence emission spectra of native OVA (curve 1) and in the presence of 50, 70 and 90% ACN (curve 2–4). (d) Emission ANS spectra of native HSA (curve 1) and in the presence of varying concentration (20–90%) of ACN (curve 2–9). Concentration of OVA was 4.44 and HSA was 3.03 µM respectively and the path length was 1 cm. Excitation wavelength for the study was 380 nm.

ANS fluorescence has been widely used as a probe to monitor the conformational transitions in proteins due to its affinity for partially exposed hydrophobic regions of protein structure [34]. Contribution of ACN, at various concentrations (0–90%) after addition of ANS, to the emission spectra was taken into account, and we report here the subtracted fluorescence spectra. Regardless of the presence of NaCl, increases in ACN concentration up to 40% produced no discernable change in ANS fluorescence intensity with OVA (Figure 3A). In the presence of 0.9% NaCl, there was a steady increase in ANS-dependent fluorescence with increasing ACN concentration from 40–90%. In the absence of NaCl, a similar trend was interrupted by an abrupt spike in ANS fluorescence reaching a maximum at 50% ACN. Above 50% ACN, ANS fluorescence diminished until ACN concentration reached 70% after which ANS-dependent fluorescence mimicked that obtained in the presence of NaCl. In a parallel experiment, OVA treated with ACN was centrifuged and the supernatant evaluated for ANS-dependent fluorescence. For OVA in the presence of NaCl, centrifugation had no effect. For OVA treated with ACN in the absence of NaCl, the spike in ANS-fluorescence at 50% ACN was still observed, but at 70% ACN and above, little if any fluorescence was detected. Data obtained in the absence of NaCl are consistent with the formation of OVA aggregates at ACN concentrations above 50%. Centrifugation removed precipitated aggregates and the ANS fluorescence due to them. Therefore, the observed increase in ANS fluorescence at 90% ACN concentration is attributable to the binding of ANS to the aggregates, rather than to partially folded states observed on folding pathway. Negligible decrease in ANS fluorescence was observed upon centrifugation of OVA treated ACN in presence of 0.9% NaCl as salts prevented the formation of aggregates.

In the presence of 0.9% NaCl, HSA showed very similar behavior to that observed for OVA with a continuous increase in relative ANS fluorescence with increasing ACN concentration regardless of whether or not the sample was centrifuged prior to data collection (Figure 3B). In the absence of NaCl, ANS fluorescence of uncentrifuged samples of ACN-treated HSA was essentially the same as that observed in the presence of NaCl. However, when samples were centrifuged and only the supernatant evaluated, there was an abrupt decrease in ANS-fluorescence above 70% ACN. The data obtained in the absence of NaCl are consistent with the accumulation of a molten globule (MG) state up to ACN concentrations of 70% followed by formation of aggregates at higher ACN concentrations. Presumably it is the pelleting of these aggregates by centrifugation that resulted in lowering of ANS fluorescence intensity [35]. In the presence of NaCl no intermediate was observed, with continuous increase in ANS fluorescence up to 90% can and centrifugation had no effect on the observed ANS-dependent fluorescence. OVA after attaining the maximum ANS fluorescence (Figure 3C) at 50% ACN (curve 2), showed a decline in the ANS fluorescence up to 70% ACN (curve 3) and after that again ascend takes place till 90% ACN addition (curve 4). This increase at 90% may be due to the formation of aggregates that ultimately results in enhanced fluorescence [23]. Binding of ANS to HSA (Figure 3D) incubated with 70% ACN produces a large boost in fluorescence intensity (∼50 times of the native, curve 7) accompanied by red shift of 2 nm from 490 nm to 492 nm indicating exposure of hydrophobic regions of the protein molecule on ACN addition. Similar increase in ANS binding to HSA has also been reported earlier indicating the exposure of more hydrophobic regions [36]. With further addition of ACN up to 90% (curve 9), there is increase in ANS fluorescence suggesting that more unfolding of protein results in binding of hydrophobic probes to form hydrophobic clusters. As increase in hydrophobicity is related to degeneration of cell, our ANS result gives an idea about the toxicity of aggregates formed [37]. In the absence of NaCl, MG states were observed at 50% and 70% ACN for OVA and HSA, respectively, and at higher ACN concentrations the protein aggregated. Maximum aggregation is attained at 90% ACN. In presence of NaCl these intermediate states were not obtained proving the effect of salting in. Moreover, presence of NaCl prevented aggregation of the protein thus, decreasing the hydrophobic environment in the vicinity of the protein. The results of ANS fluorescence are consistent with intrinsic fluorescence.

### Attenuated Total Reflection Fourier Transformed Infrared Spectroscopy (ATR-FTIR)

**Figure 4:**
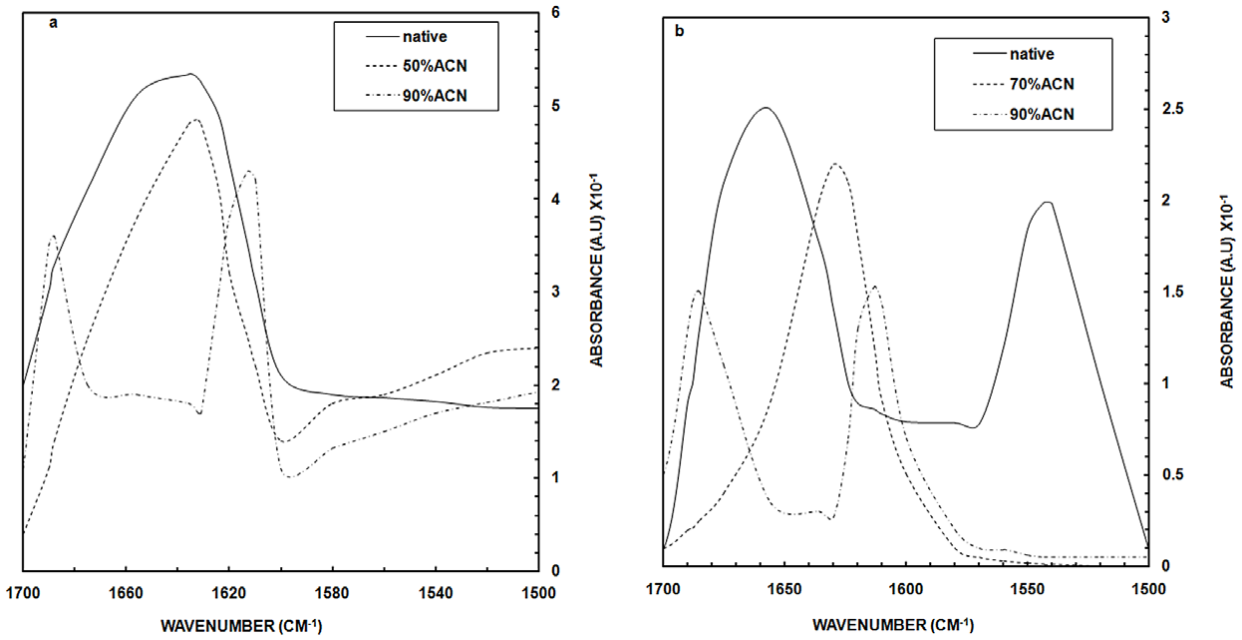
FTIR Analysis. ATR-FTIR spectra of (a) OVA (b) HSA with varying concentration of ACN in the amide I region in the range of 1720 to 1580 cm-1. The spectra were obtained with the proteins at concentration of 44.4 µM for OVA and 30.3 µM for HSA in 20 mM phosphate buffer, pH 7.2.

Infrared spectroscopy identifies β-strands more effectively than α-helices [38]. The conformational changes observed in OVA on addition of ACN were evaluated by monitoring amide I band FTIR spectra (Figure 4A). In the absence of ACN, OVA showed a broad peak around 1636–1656 cm^−1^ indicating the presence of α-helices and β-strands, consistent with its known structure [39]. Little if any change in spectrum was observed on addition of ACN up to 40% (data not shown). At 50% ACN, a peak was observed at 1632 cm^−1^ suggesting a preponderance of β-sheet structure associated by way of *intra*molecular hydrogen bonding. At 90% ACN, two distinct bands (1688 and 1613 cm^−1^) were suggestive of development of *inter*molecular hydrogen bonds that results in coagulation of protein molecules. The appearance of these peaks in the amide I region implies that the aggregated species possess extensive β-sheet structure. This indicates that there is a progressive increase in β-sheet configuration leading to the formation of protein aggregates. Importantly, this type of infrared spectrum has been shown to be characteristic of amyloid fibrils [9], [40].

In the absence of ACN, HSA showed peaks in the amide I region at 1656 (mainly C = O stretch) and an amide II region peak at 1543 cm^−1^ (C-N stretching coupled with N-H bending modes) confirming its predominantly helical structure (Figure 4B). Upon incubation of HSA with 70% ACN for four hours, there was a substantial shift in the peak to 1630 cm^−1^, suggesting the appearance of a much greater proportion of β-sheet structure bonded by i*ntra*molecular hydrogen bond. In the presence of 90% ACN, there was a complete loss of the transitions at 1543 cm^−1^ and 1656 cm^−1^ along with the emergence of new features at 1616 and 1688 cm^−1^. As above, this suggests a transition from intramolecular hydrogen bonded to intermolecular hydrogen bonded β-sheet structure [41]. Reduction of α-helical structure of HSA caused the partially unfolding of protein, followed by formation of a state with higher β-sheet than α-helical structure contents. A similar α- helix to β-sheet transition has also been reported in polylysine and prion protein [8]. These data suggest the formation of intermolecular hydrogen bonded β-sheets, providing one mechanism by which HSA may aggregate at 90% ACN.

### Far-UV Circular Dichroism (CD) Studies

**Figure 5:**
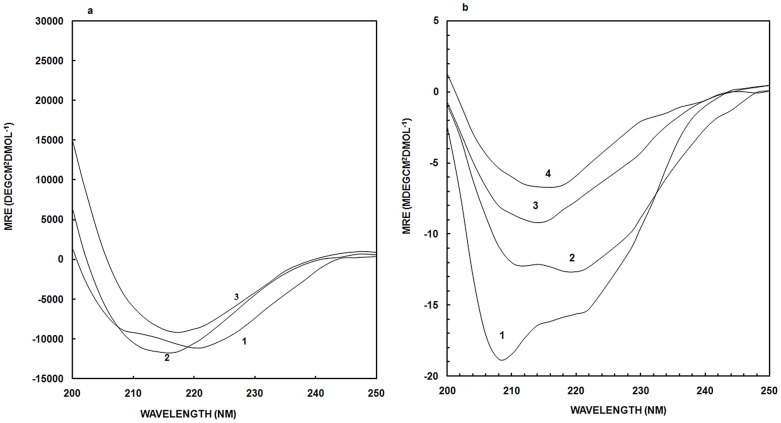
CD studies. (a) Far-UV CD spectra of OVA in the presence of ACN in 20 mM sodium phosphate buffer, pH 7.2. Curve 1 shows native OVA, curve 2 and 3 corresponds to OVA at 50% and 90% ACN respectively; (b) Far-UV CD spectra of HSA in the presence of ACN in 20 mM sodium phosphate buffer, pH 7.2. Curve 1 shows native HSA, curve 2 represents HSA in presence of 40% ACN; curve 3 and 4 represents HSA at 70% and 90% ACN respectively. Concentration of OVA in the samples was 4.44 and HSA was 3.03 µM respectively and the path length was 0.1 cm.

Figure 5A depicts the far-UV CD spectra in the 250-190 nm range of native OVA (curve 1) with the minima at 222 and 208 nm indicating the presence of α-helix structure. Curve 2 represents ACN-induced state of OVA at 50% resulting in loss of the feature at 222 nm and emergence of a new signal at 217 nm characteristic for the β-sheet conformation [42]. This indicates a loss in α- helix content with the induction of β-sheet conformation [39]. On further addition of ACN up to 90% (curve 3), the β-sheet structure is enhanced, with retention of peak at 217 nm. This result also confirms, in agreement with the FTIR spectra, that at 90% ACN in OVA most of the α-helix structure has been converted to β-sheet form. The changes in OVA secondary structure induced by ACN were evaluated by monitoring mean residue ellipticity at 222 nm (Figure S1). As can be easily seen in the figure, initially structural loss has been taking place up to 20% ACN addition. But afterwards there is gradual increase in secondary structure of the protein up till 50% ACN, further addition of ACN results in structural loss.

Similarly, Figure 5B shows the far-UV CD spectra of native HSA and in the presence of ACN. The spectra of native HSA shows minima at 208 and 222 nm, characteristic of helical structure (curve 1). From native to ACN-induced intermediate state, transition occurs in the vicinity of 40% (curve 2), reflecting the loss of secondary structure. At 70% ACN concentration (curve 3), decrease in negative MRE, [θ] at 222 nm, occurs as a consequence of changes in protein structure originating from the formation of an intermediate state. The MRE of CD parameter at 222 nm is proportional to the α-helix secondary structure; therefore, decrease in this value is an indication of the reduction of α-helix content of the protein. On further addition of ACN, up to 90% (curve 4), complete loss of peak at 208 and 222 nm along with emergence of new peak at 216 nm, indicating the formation of β-sheet structure, most probably originating from the fibrillar aggregates in solution [32]. HSA is known to form aggregates at high temperature and low pH [13]. Changes in far-UV CD spectra of HSA (Figure 5B) were more pronounced than of OVA (Figure 5A) with the loss of more α-helix in HSA than in OVA. For better resolution, the CD parameter MRE at 208 nm is plotted as a function of increasing concentration of ACN (Figure S2). As can be seen from the figure, loss of α-helix in HSA occurs at 40% and reduces to less than half at 70% ACN. Finally, at 90% ACN, HSA retains only about one-fourth of the native α-helix content. From these observations, it can be concluded that HSA in presence of high concentration of ACN (70% and above) loses α-helix and is subjected to conformational changes relative to native physiological structure.

### Rayleigh Scattering Measurements

**Figure 6:**
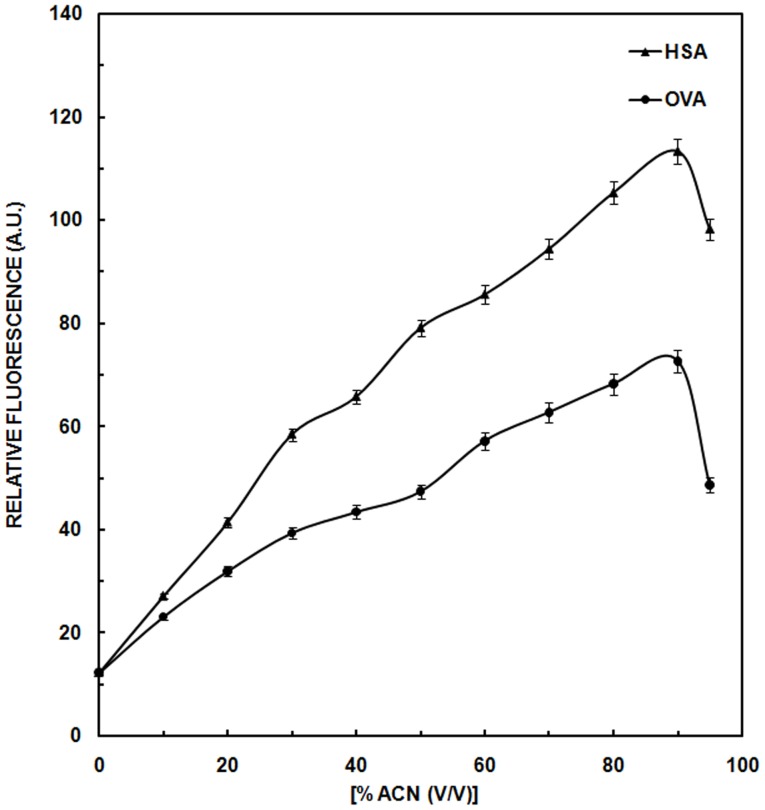
Rayleigh scattering measurements. OVA and HSA in the presence of 90% ACN. The excitation wavelength was 350 nm and emission was recorded in the wavelength range of 300–400 nm. Path length used was 1 cm and the protein concentration in the samples was 4.44 µM for OVA and 3.03 µM for HSA.

As measurement of fluorescence intensity at 350 nm is an excellent approach to check protein aggregates, light scattering of OVA and HSA in the presence of ACN (0–95%) was monitored. A six folds increase in fluorescence intensity was observed in OVA whereas in HSA at 90% ACN ∼ nine folds enhancement in fluorescence intensity (Figure 6) was observed. Thus, suggesting the formation of aggregates in albumins [43] at this concentration of ACN.

Absorbance was also monitored at 280 nm on UV-vis spectrophotometer and respective absorbance values were plotted as a function of increasing concentration of ACN (Fig. S3). These results depicted there is no loss in concentration of OVA and HSA upon incubation with different concentration of ACN. Thus ruling out the possibility of protein loss/concentration error and the effect is purely an aggregation effect.

### Thioflavin T Analysis

**Figure 7:**
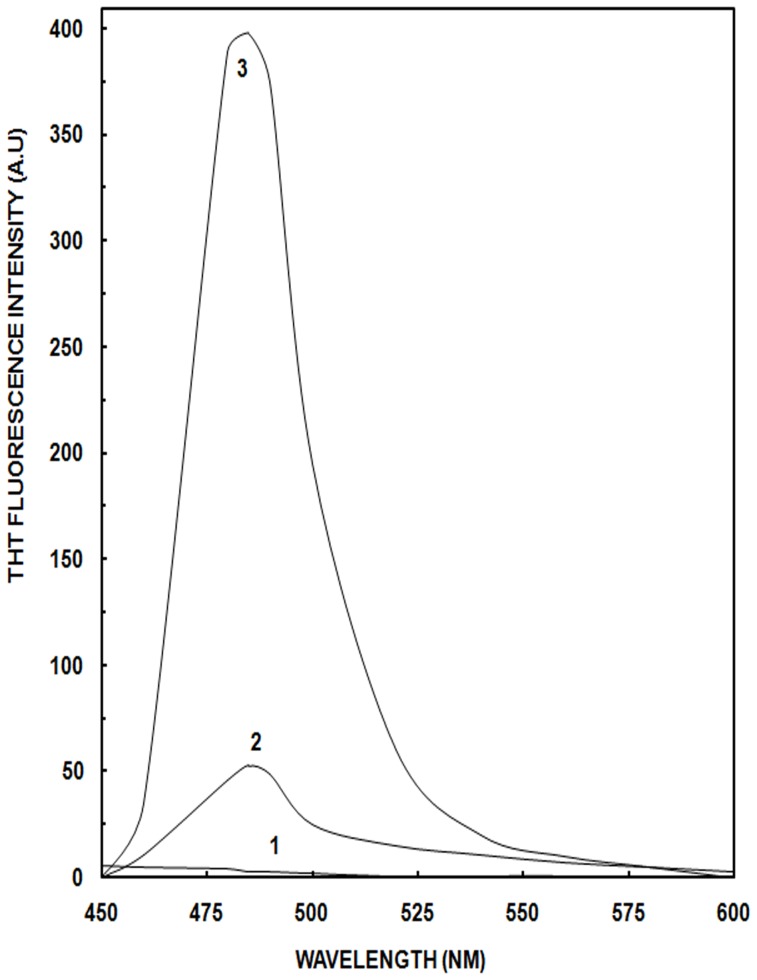
Thioflavin T assay. Thioflavin T spectra of HSA in the presence of ACN. Curve 1 shows native protein; curves 2 and 3 represent HSA at 70% and 90% ACN respectively. Thioflavin T fluorescence was monitored at an excitation wavelength of 440 nm. Concentration of OVA and HSA for the analysis was 4.44 and 3.03 µM and the path length was 1 cm.

To further analyze that the ordered β-sheet structure and exposed hydrophobic surface of OVA and HSA at 90% ACN results in aggregation, Thioflavin T (Th T) assay was performed. Th T is the most commonly used dye to diagnose amyloid fibril formation, both in vivo and in vitro [26]–[27]. The binding of ACN (0–90%) to ThT dye was taken into account and here we reported the subtracted spectra. Th T spectra were recorded for the HSA and OVA with varying concentration of ACN (0% to 90%) incubated for time intervals of 4, 6, 8, 12, 24 and 48 hours after addition of Th T dye to them. Spectra of all the samples except 80% and 90% ACN of albumins obtained at 4, 6, 8 and 12 hours intervals did not show any prominent peak indicating that there is no aggregate formation at these time intervals. Even spectra at 80% and 90% ACN showed negligible fluorescence intensity (data not shown). Furthermore, when spectra were recorded after 24 hours incubation, prominent increase in fluorescence intensity was detected in albumins at 90% ACN, indicating the formation of aggregates. In addition to this, on increasing the incubation time to 48 hours (data not shown), results similar to 24 hours were observed confirming the binding of Th T dye to aggregates and attainment of equilibrium. Figure 7 depicts Th T spectra of HSA at different concentrations of ACN incubated for 24 hours. Native HSA (curve 1) show no binding of the dye. At 70% ACN, 8 times Th T fluorescence enhancement relative to native was observed (curve 2). The maximum Th T fluorescence of about 133 times in HSA was observed at 90% ACN (curve 3). From these observations we conclude that the formation of extensive β-sheet aggregates with exposure of side chain residues of HSA at 90% ACN, leads to steric interaction between these residues and Th T dye, ultimately results in high fluorescence. Figure S4 depicts relative Th T fluorescence intensity of HSA and OVA incubated with varying concentrations of ACN. Maximum aggregate formation in albumins was observed at 90% ACN. HSA shows more aggregates formation in comparison to OVA as can be interpreted by the relatively high Th T fluorescence in HSA than OVA.

### Congo Red Assay

**Figure 8:**
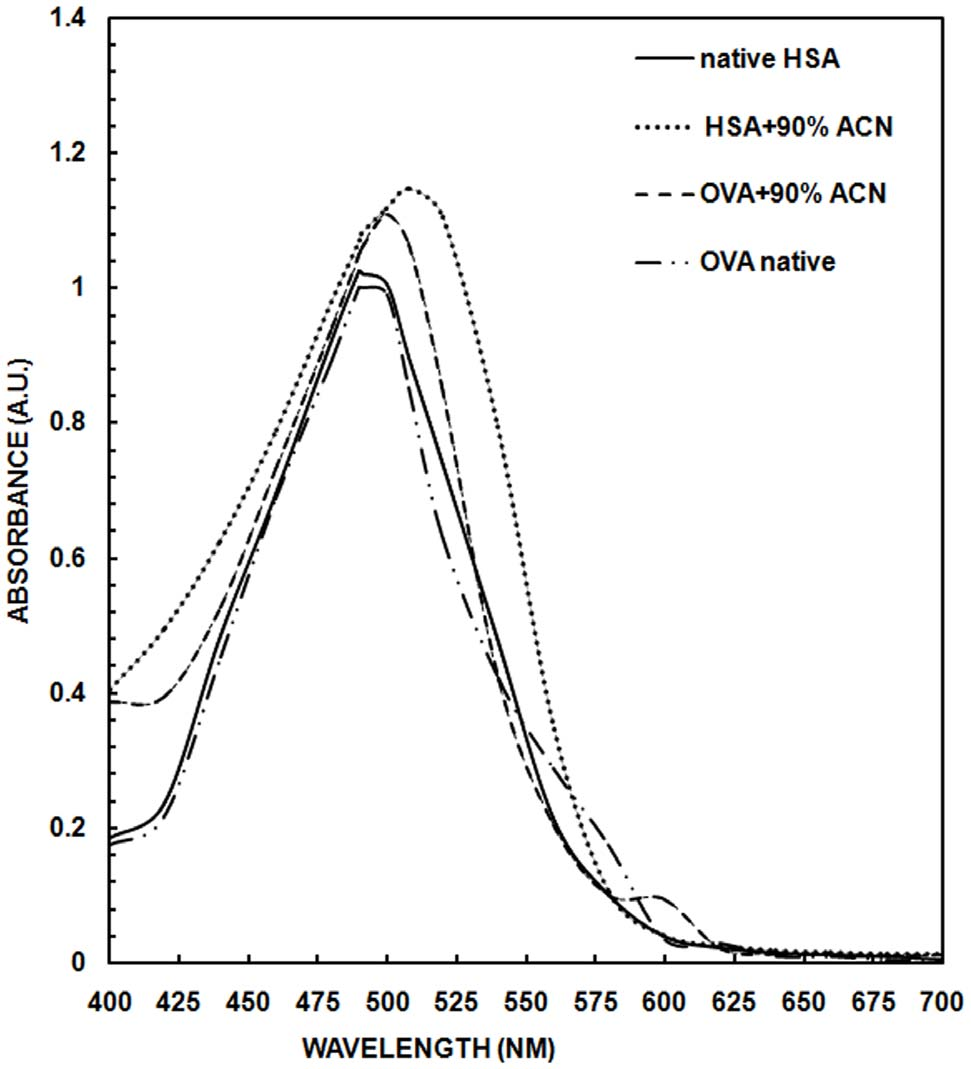
Congo red assay of albumins. Congo red dye binding of OVA and HSA in the presence of 90% ACN. Concentration of OVA and HSA was 4.44 and 3.03 µM respectively and the path length was 1 cm. Absorbance was recorded in the range of 400 to 700 nm.

The azo dye CR has a high affinity with the β-pleated structure of all forms of amyloid. The repeating β-sheet structure allows the hydrophobic dye like CR to interact with regularly spaced protein chains, which is commonly used to monitor amyloid formation in-vitro. The interaction between CR and protein is due to the electrostatic interaction between sulphonic group of CR and positively charged amino acids of protein [27]. As shown in Figure 8, both native HSA and OVA showed peak at 490 nm. At 90% ACN, a red shift of 18 and 10 nm with prominent increase in absorbance was observed in HSA and OVA respectively. The absorbance peak was observed at 508 nm in HSA whereas at 500 nm in OVA.

### Dynamic Light Scattering (DLS) Measurements

To confirm the presence of albumin aggregates at 90% ACN, DLS studies were performed. Hydrodynamic radii (*R*_h_) and percent polydispersity (% P*d*) of native as well as 90% ACN treated HSA and OVA have been calculated in Table 2. Native HSA and OVA shows R*h* of 3.5 and 3.0 nm whereas 19.4 and 11.5% P*d*. Upon incubation with 90% ACN, both the albumins showed an increase in R*h,* % P*d* and apparent molecular weight confirming aggregate formation. Here R_h_ values are in agreement with the prior observations [44]–[45]. Lower P*d* values show the occurrence of homogenous environment and lack of any association-dissociation process, thereby confirming monomeric nature of native HSA and OVA. Increase in R_h_ and apparent molecular weight signify the presence of aggregated assemblies due to reduction in intramolecular interactions in the presence of ACN. This results in the exposure of hydrophobic residue which can, now, interact among them to form intermolecular interactions and hence, aggregates of albumins. Here, increase in P*d* of the solution further confirms the presence of heterogeneous species in the solution.

**Table 2:** Hydrodynamic radii (R_h_) and polydispersity (P*d*) of HSA and OVA in the absence and presence of ACN.

S.No.	Condition	Hydrodynamic Radii (nm)	Polydispersity (%)	Apparent Molecular weight (kDa)
**1**	Native HSA	3.5	19.4	68
**2**	Native OVA	3.0	11.5	44
**3**	HSA+90% ACN	6.1, 91.3	27.2, 51.1	109, 185213
**4**	OVA+90% ACN	4.8, 88.4	24.5, 48.0	83, 174856

### X-ray Diffraction Analysis

**Figure 9:**
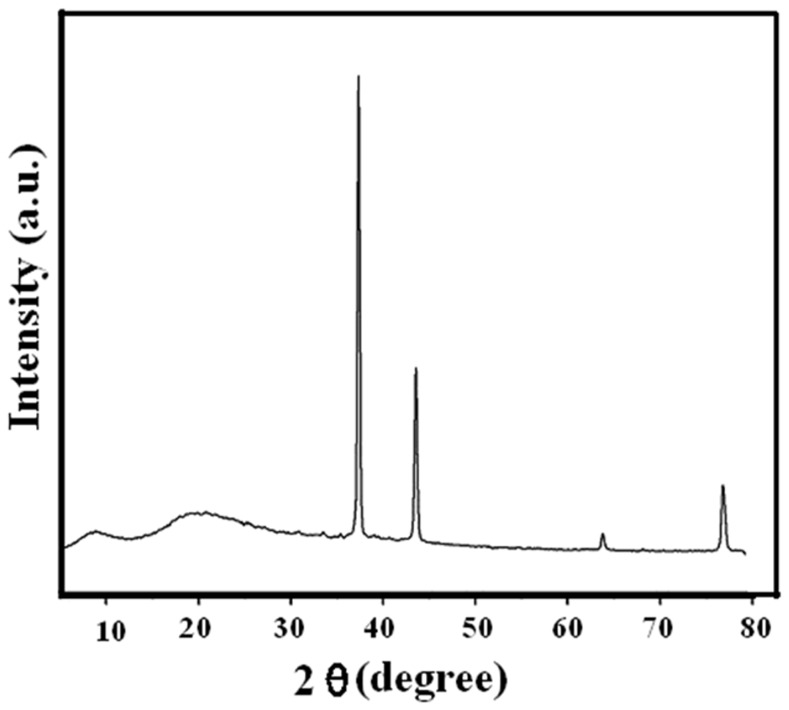
XRD pattern of HSA aggregates in presence of 90% ACN. The concentration of HSA was 10 mg/ml.

XRD of HSA incubated with ACN is shown in Figure 9 as a plot of scattering intensity vs. the scattering angle 2θ. Two strong reflections can be observed: a dominant sharp and intense reflection occurs at a diffraction angle of 37°, and one weaker, more diffuse, but still intense reflection is observed at ∼44°. These peaks may be due to the formation of HSA aggregates. Emergence of slight peak around the diffraction angle of 80° indicates the presence of fibril aggregates that precede formation of amyloid fibril [13], [46].

### Effect of Aggregated HSA on DNA Breakage in Lymphocyte

**Figure 10:**
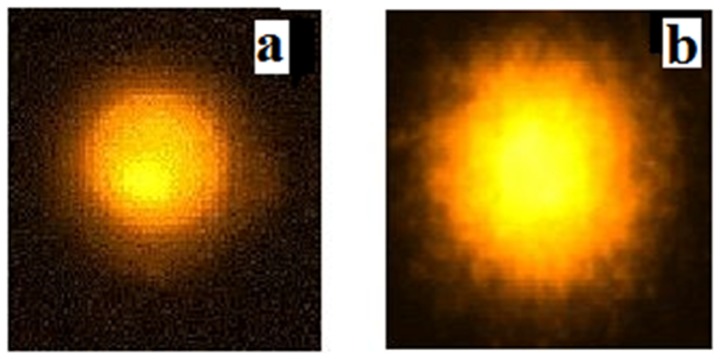
SCGE assay. Images of plasmid DNA damage in native HSA treated lymphocytes (a) and lymphocytes treated with HSA aggregates (b). The concentration of aggregates was 50 µg/ml.

Figure 10 demonstrates the Single Cell Gel Electophoresis (SCGE). Figure 10A and 10B represents the native HSA incubated with lymphocytes and nuclear DNA damage in lymphocytes by aggregated HSA respectively. Negative control (without any treatment) and positive control (3 µl of methyl methane sulfonate (25 µg/ml)) treatments are shown in Figure S5A and S5B respectively. ACN was air dried before adding aggregated HSA to lymphocytes; it causes nuclear DNA breakage of about 12 µm tail length compared to 4 µm tail length of negative control and 20 µm tail length of positive control. This image clearly demonstrates that aggregated HSA cause cell necrosis and has a genotoxic effect on lymphocytes in vitro. On contrary, in native HSA tail length of 6 µm was observed very close to negative control. Damage in lymphocytes in presence of aggregated HSA may be attributed to the fact that the DNA damage is caused by two major mechanisms, free radical reactions and direct binding to DNA. The fibrillar aggregates that precede formation of mature amyloid fibrils may be the primary toxic species. This toxicity is likely to arise because in these early aggregates hydrophobic side chains and other regions of the polypeptide chain will be much more accessible than in the fully formed mature fibrils. Indeed, the latter are often found to be remarkably inert, for example in their resistance to proteolysis and degradation [47]. It has been demonstrated that intracellular protein aggregation directly causes free radical production [48]. Interestingly, Tabner and colleagues showed that there is a tight correlation between the time of oligomer formation and a ROS burst during such reactions, suggesting that it is the early aggregation steps and not the production of amyloid fibrillar structures that may be associated with free radical production [49].

### Scanning Electron Microscopy Analysis

**Figure 11:**
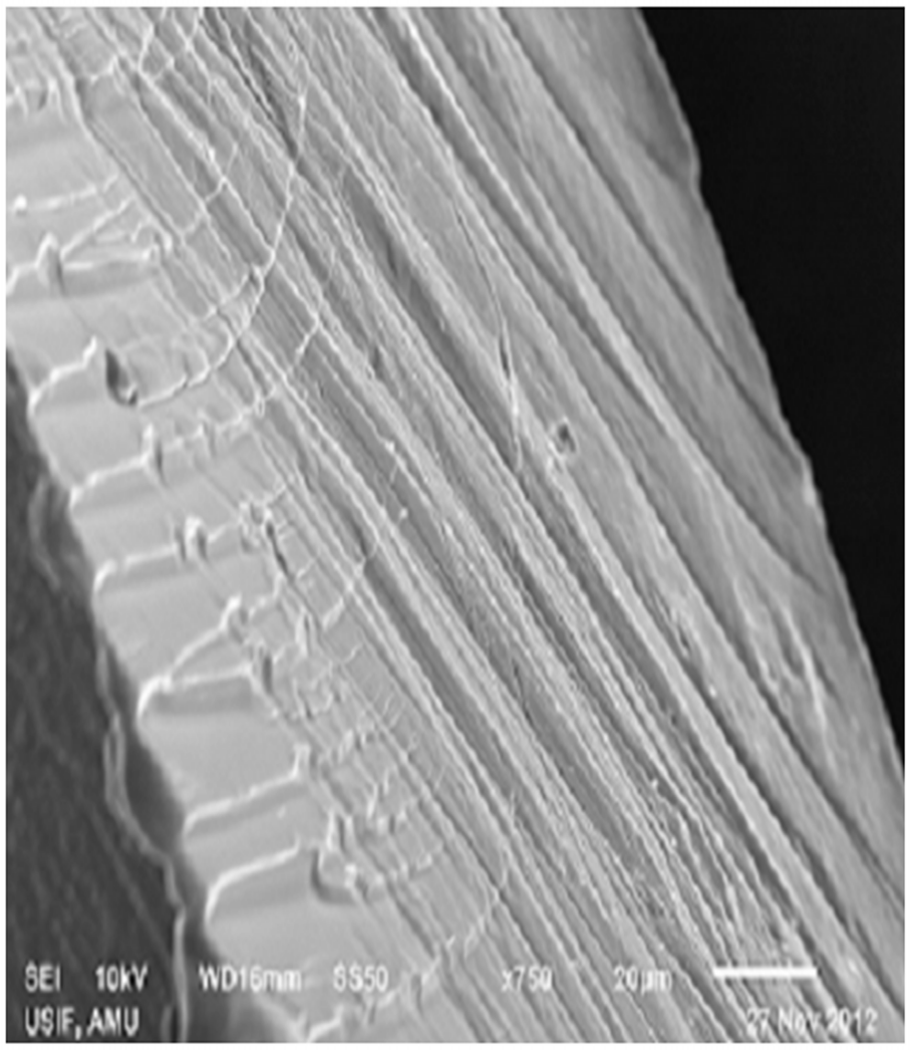
SEM analysis. HSA aggregate formed in presence of 90% ACN. Concentration of HSA was 10 mg/ml.

The SEM is used to generate high-resolution images of protein aggregates and amyloid fibrils. HSA incubated with 90% ACN clearly shows the formation of fibrillar aggregates (Figure 11). Also, images in Figure S6A and S6B show these altered HSA molecules structures leading to the formation of aggregates. Figure S6C represents the formation of amorphous OVA aggregates. The growth of this aggregate formation is dependent on the interaction between these fibrillar structures which are responsible for the formation of supra-fibrillar [13].

### Conclusions

**Figure 12:**
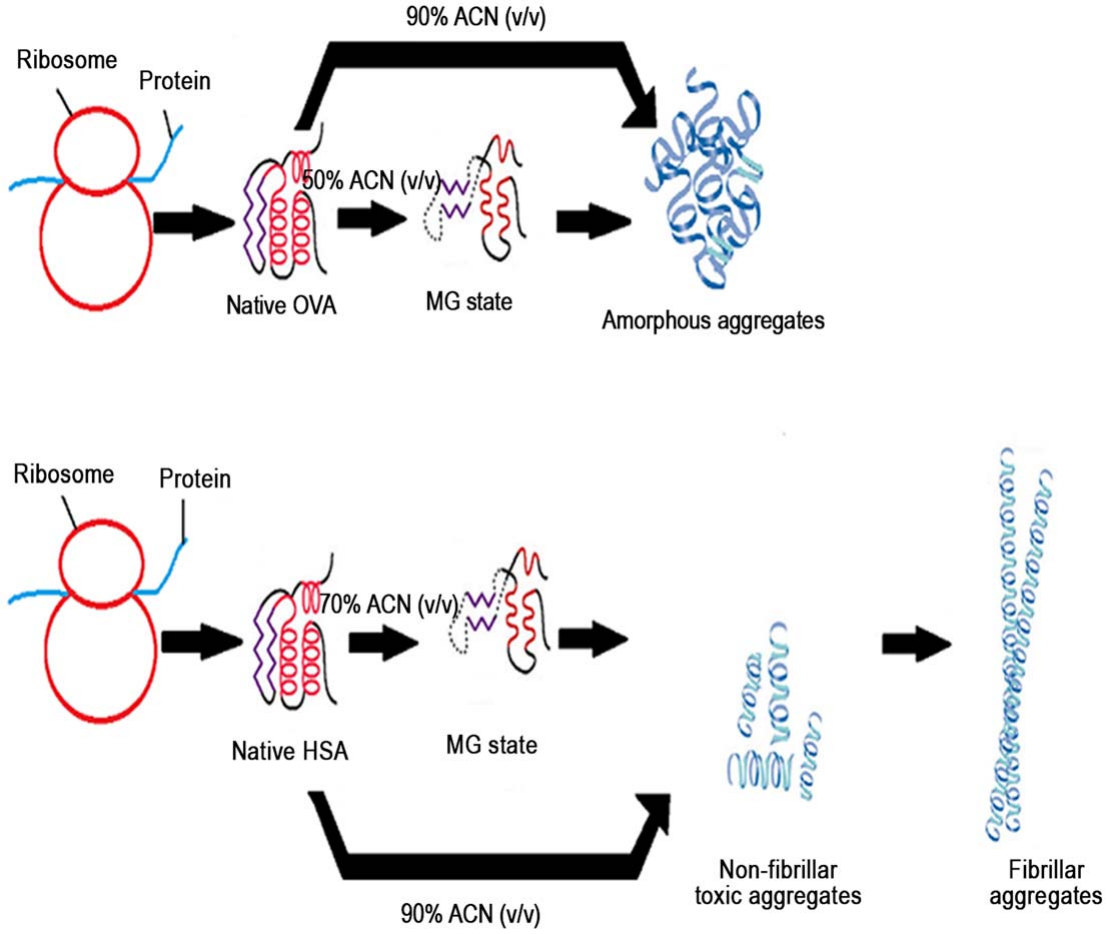
Outline of the work. Schematic representation of albumin structural alteration pathway in the presence of ACN.

Recently it has been thoroughly shown that protein sequences/structures themselves have specific biases working against misfolded conformations. The interactions between the misfolded states results in aggregation or non-native structures [50]. In this study, ACN is used as a solvent for the formation of albumin amyloid fibrils in vitro. However, from this study, the negative effect of ACN on human population cannot be proved. When these results were taken together, i.e. high ANS fluorescence, retention of secondary structure in OVA at 50% ACN and in HSA at 70% ACN suggests that this state resemble the MG state as defined for other proteins. Identification of the structural characteristics of these partially folded states is important for understanding the pathway of protein folding. Further incubation of albumins at high concentration of ACN (at physiological pH), i.e. 90%, result in the formation of aggregates as confirmed by Thioflavin T and Congo red analysis. HSA aggregates were further confirmed as fibrillar in nature by XRD and SEM studies. Carrying our studies to lymphocyctes, we have found that the HSA aggregates were genotoxic for cell. Overall pathway of modification of albumins as a result ACN addition has been shown in Figure 12 for easy understanding. The misfolding and aggregation of proteins is a very common phenomenon both in the cell, *in vitro* protein refolding, and the corresponding biotechnological applications. Our work will facilitate further understanding the genotoxicity of aggregated protein and conformational changes in the presence of organic solvent and can prove to be very useful for investigating the molecular basis of diseases due to the formation of aggregates.

## Supporting Information

Figure S1 **Relative CD study.** Relative intensity of Far-UV CD at 222 nm for OVA as a function of varying concentration of ACN.
(TIF)

Figure S2 **Relative CD study.** Relative Far-UV CD intensity of HSA at 208 nm as a function of varying concentration of ACN.
(TIF)

Figure S3 **Absorption study.** Absorbance of albumins at 280 nm as a varying concentration of ACN. Final concentration of HSA and OVA was 3.03 and 4.44 µM. All the reactions were carried out at 37°C.
(TIF)

Figure S4 **Thioflavin T fluorescence study.** Relative ThT fluorescence intensity of HSA and OVA as a function of varying concentration of ACN.
(TIF)

Figure S5 **SCGE assay.** Images of lymphocytes nuclei damage in negative control (a) and in lymphocytes with positive control (b).
(TIF)

Figure S6 **SEM analysis.** Images of aggregated HSA (a & b) and OVA (c) in the presence of 90% ACN.
(TIF)
