# Correction: Deciphering Structural Intermediates and Genotoxic Fibrillar Aggregates of Albumins: A Molecular Mechanism Underlying for Degenerative Diseases

**DOI:** 10.1371/annotation/04fa421f-3dfe-466a-8c21-17a7bab1494c

**Published:** 2013-11-15

**Authors:** Aabgeena Naeem, Samreen Amani

There are errors in the legend for Figure 10. The correct figure legend is: SCGE assay: Images of lymphocyte genomic DNA damage in the presence of (a) native HSA and (b) HSA aggregates. The concentration of native HSA and HSA aggregates was 50 mg/ml.

There are errors in Table 1. In row 1, HAS should be HSA. In row 3 and 4 CAN should be ACN.

