# Supplementary figures and images for: Deciphering Structural Intermediates and Genotoxic Fibrillar Aggregates of Albumins: A Molecular Mechanism Underlying for Degenerative Diseases
Source: PLoS One. 2013 Jan 14;8(1):e54061. doi: 10.1371/journal.pone.0054061 (PMC3544675; doi:10.1371/journal.pone.0054061)

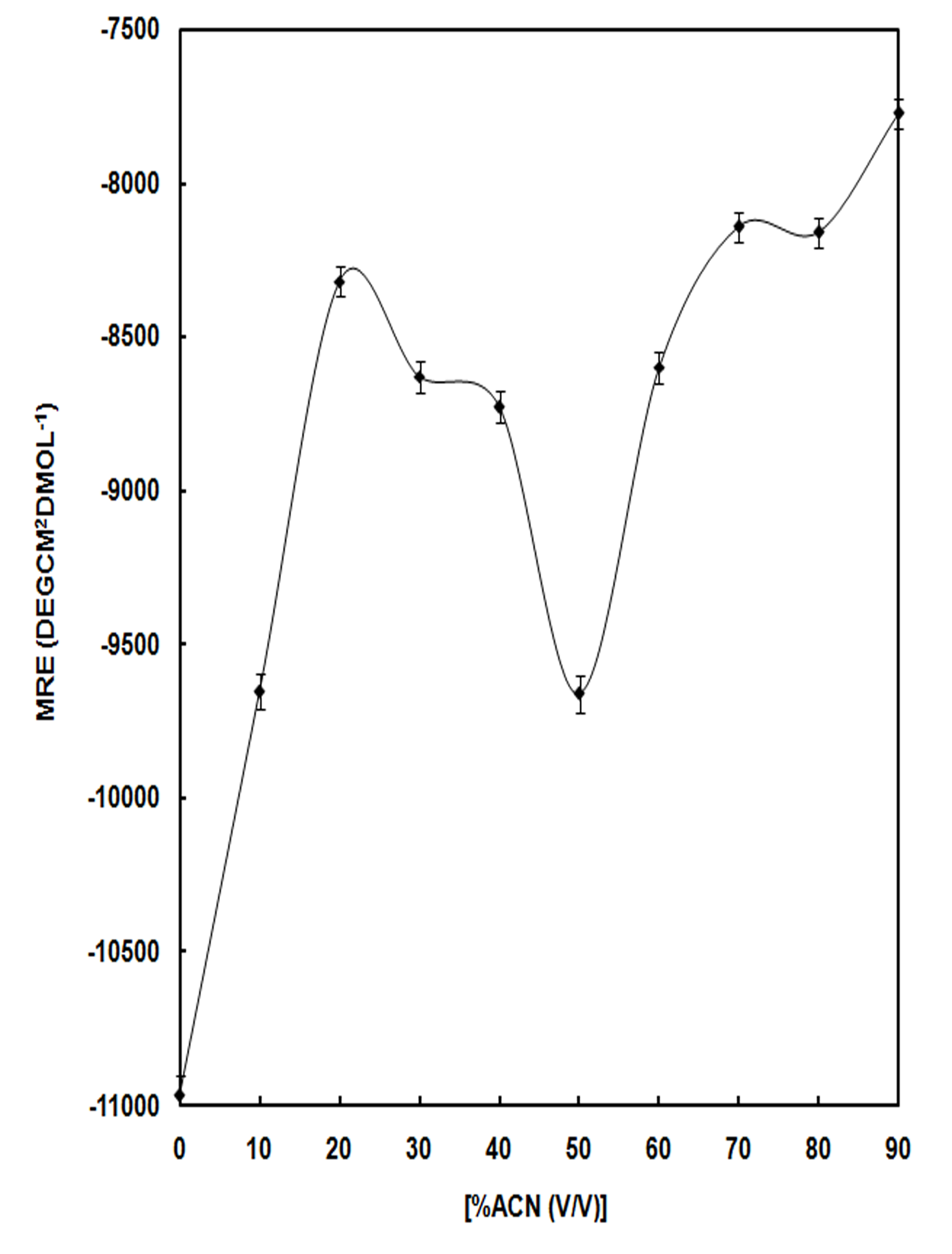

Supplement: Figure S1 — Relative CD study. Relative intensity of Far-UV CD at 222 nm for OVA as a function of varying concentration of ACN. (TIF) [file pone.0054061.s001.tif]

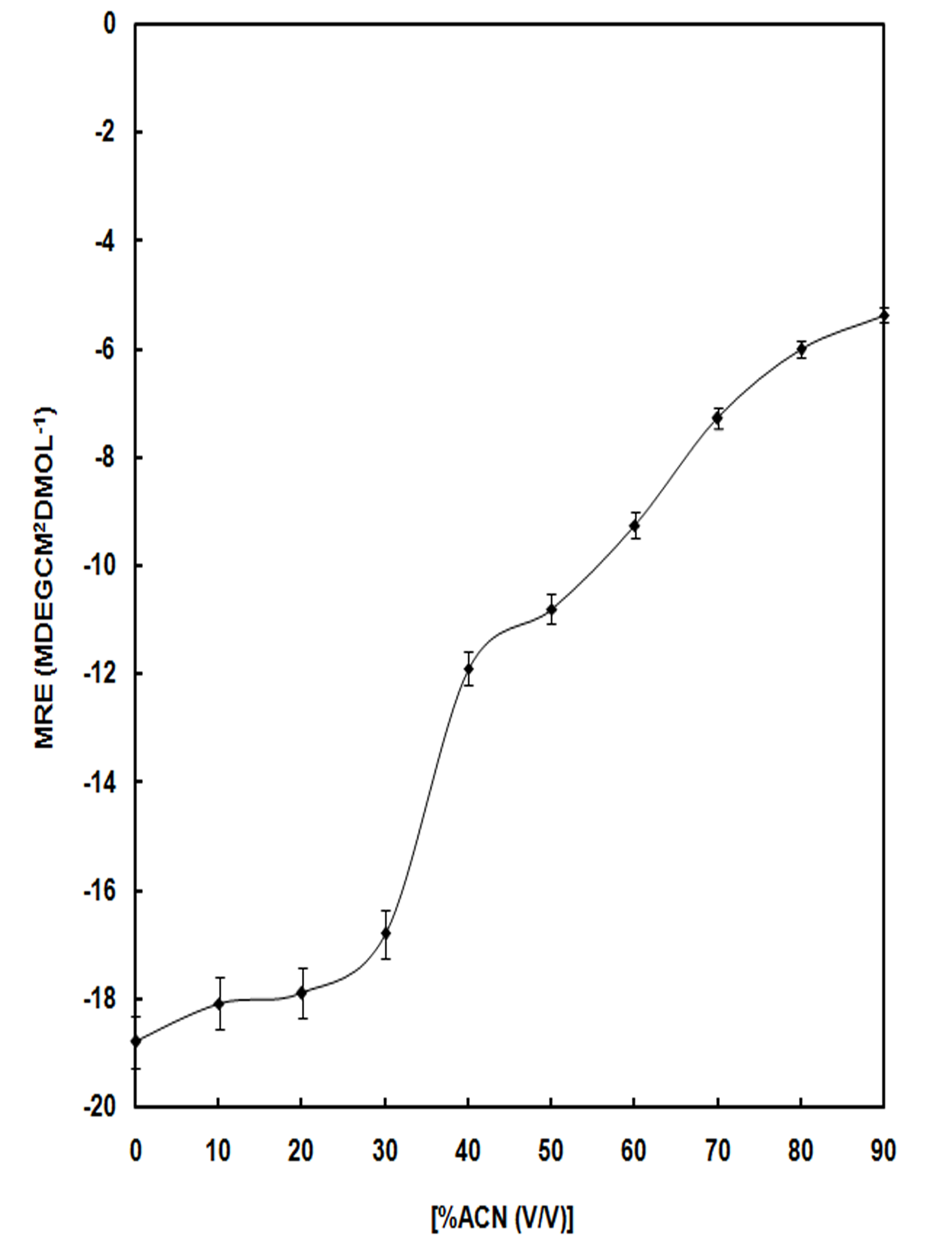

Supplement: Figure S2 — Relative CD study. Relative Far-UV CD intensity of HSA at 208 nm as a function of varying concentration of ACN. (TIF) [file pone.0054061.s002.tif]

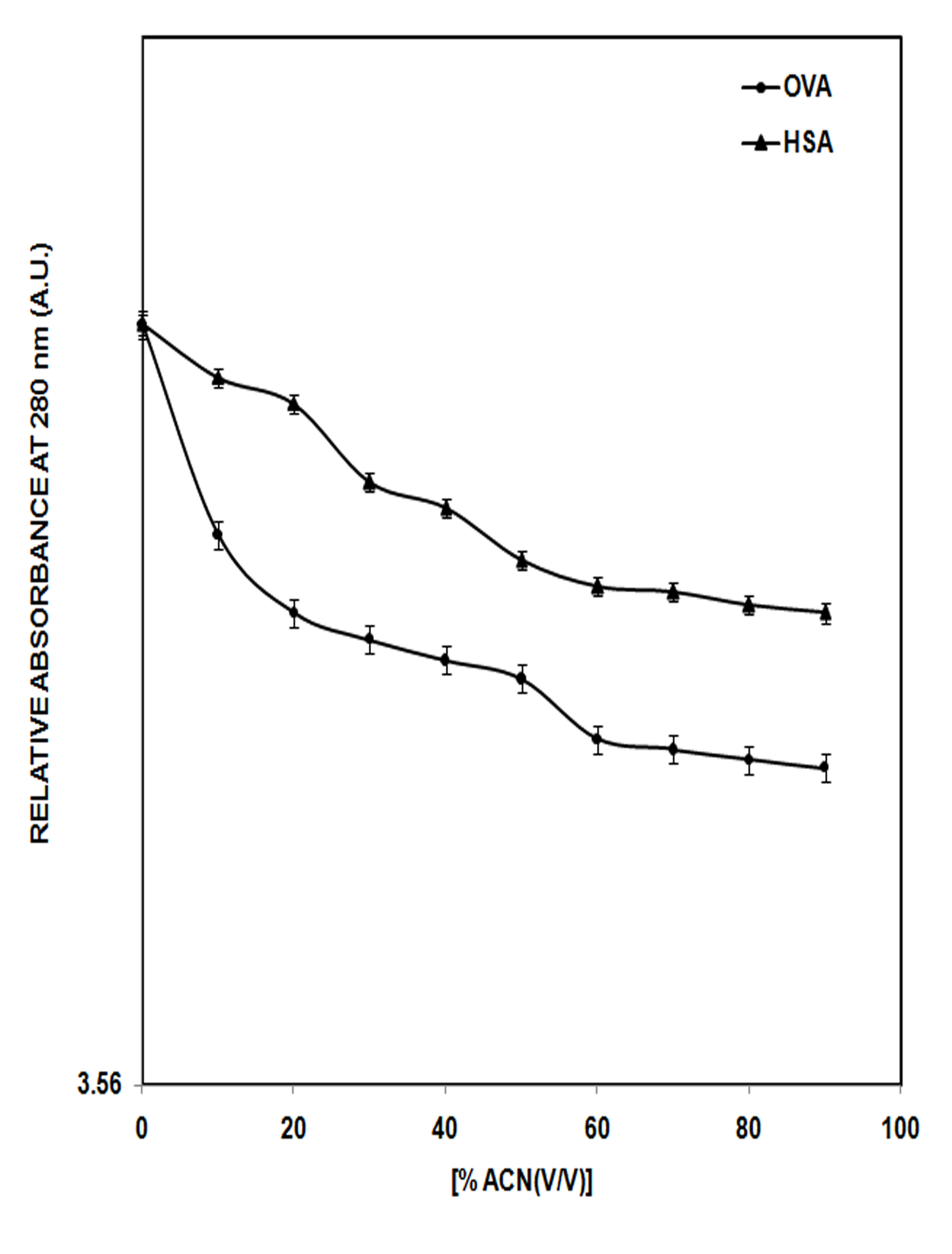

Supplement: Figure S3 — Absorption study. Absorbance of albumins at 280 nm as a varying concentration of ACN. Final concentration of HSA and OVA was 3.03 and 4.44 µM. All the reactions were carried out at 37°C. (TIF) [file pone.0054061.s003.tif]

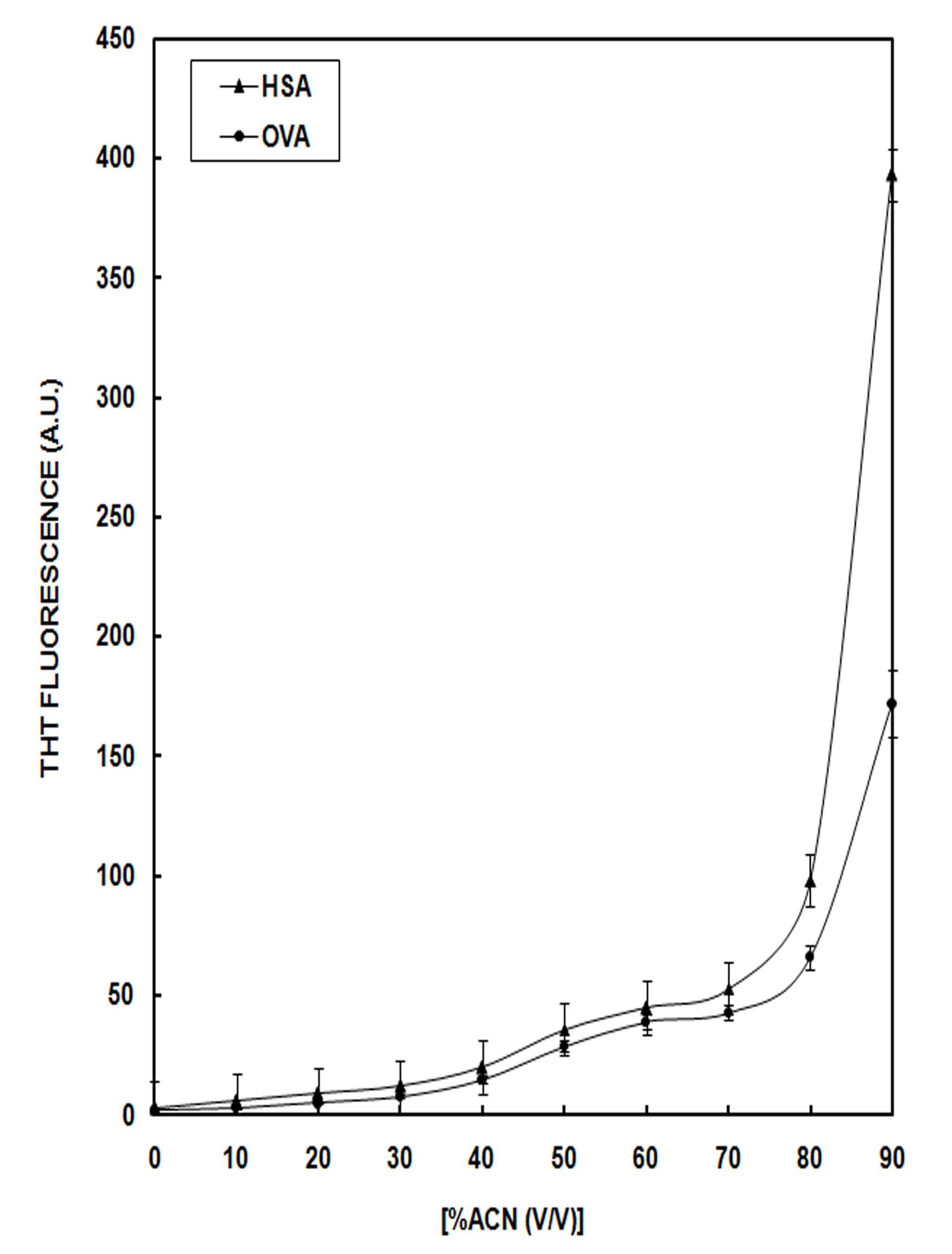

Supplement: Figure S4 — Thioflavin T fluorescence study. Relative ThT fluorescence intensity of HSA and OVA as a function of varying concentration of ACN. (TIF) [file pone.0054061.s004.tif]

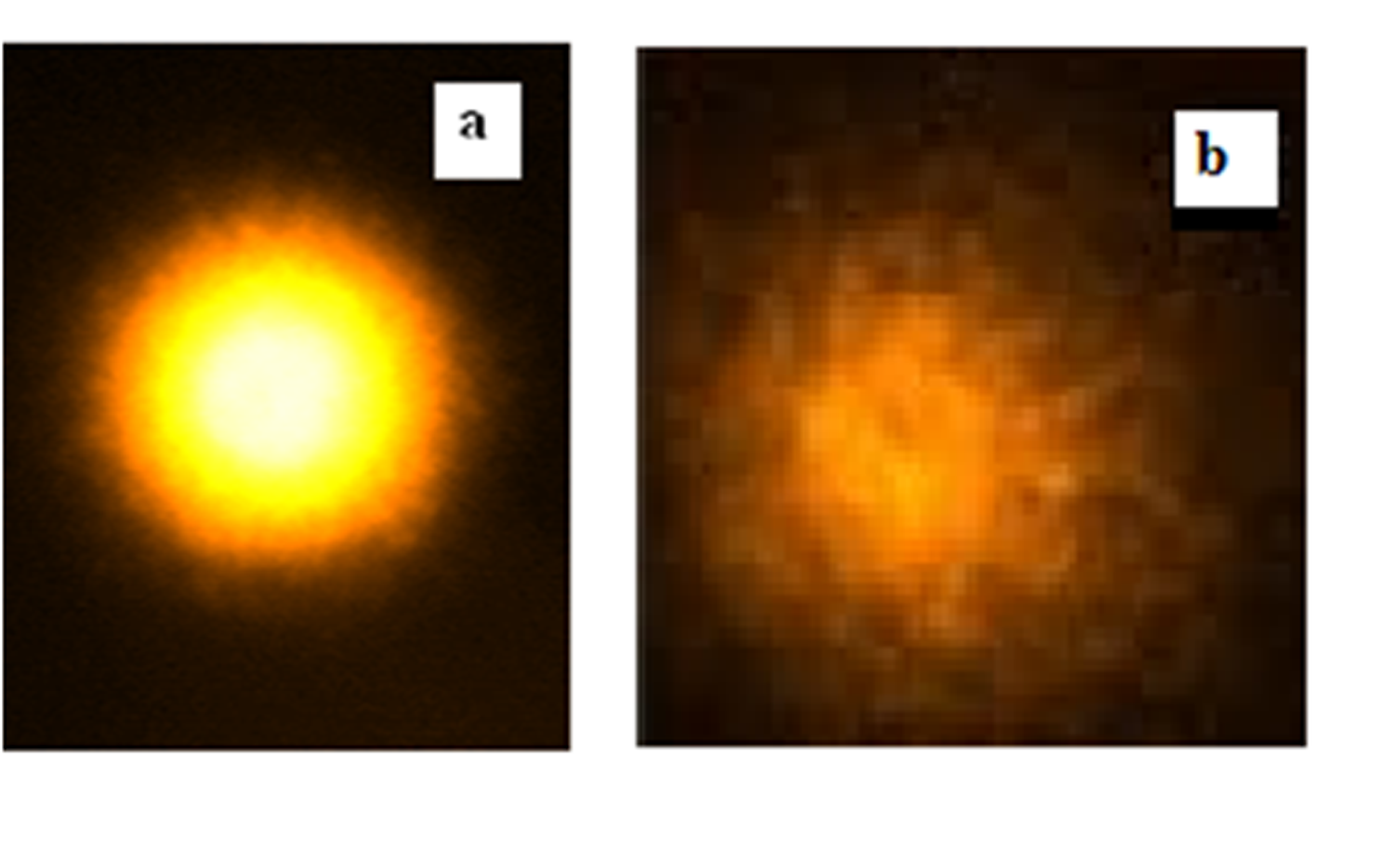

Supplement: Figure S5 — SCGE assay. Images of lymphocytes nuclei damage in negative control (a) and in lymphocytes with positive control (b). (TIF) [file pone.0054061.s005.tif]

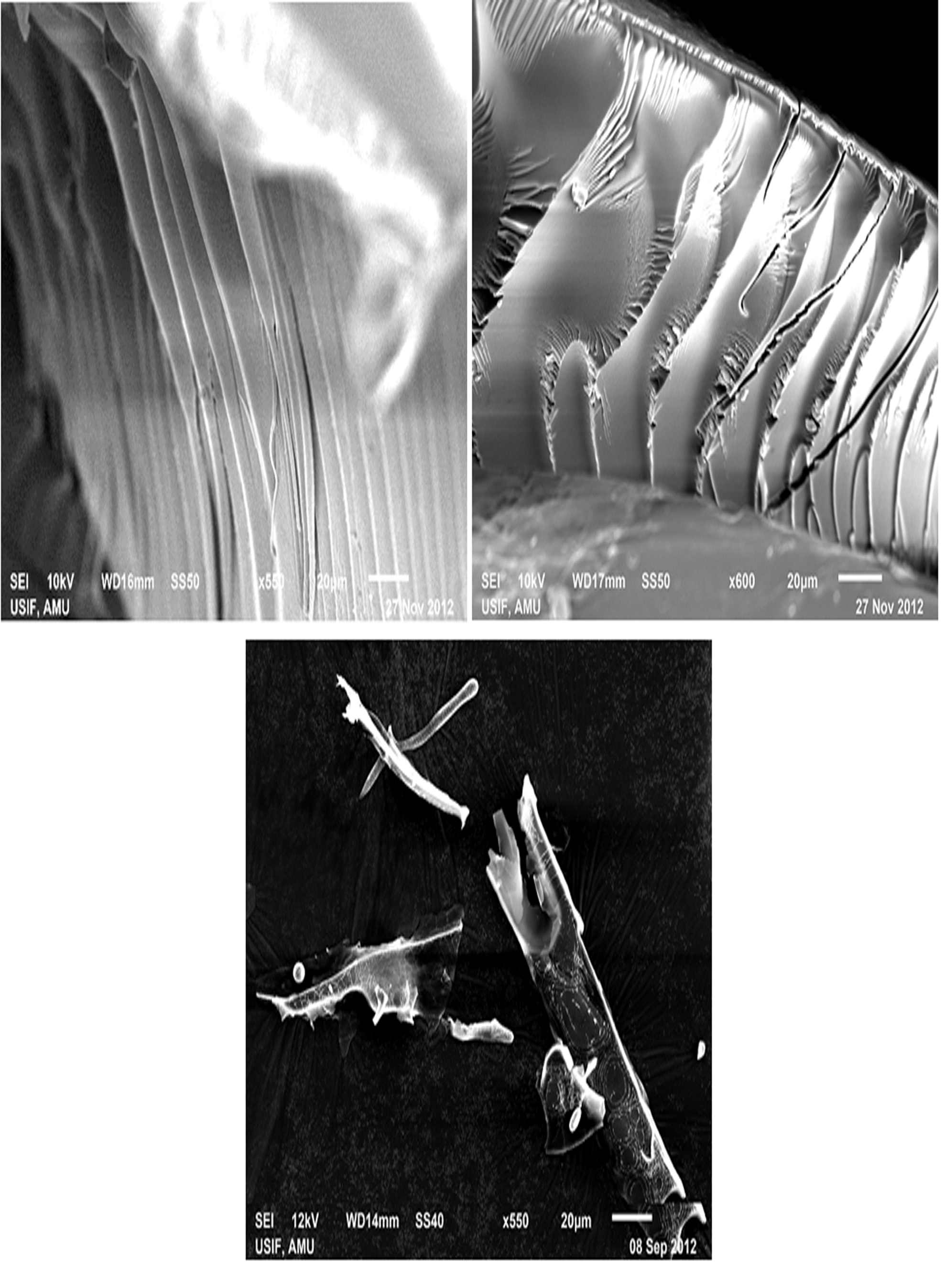

Supplement: Figure S6 — SEM analysis. Images of aggregated HSA (a & b) and OVA (c) in the presence of 90% ACN. (TIF) [file pone.0054061.s006.tif]
